# Advantages and Disadvantages of Reduced Silver in Dental Therapeutics*Read before the Michigan State Dental Society, April 7-11, 1919, and published in *Journal N. D. A*.

**Published:** 1919-11

**Authors:** U. Garfield Rickert

**Affiliations:** Ann Arbor, Mich.


### ADVANTAGES AND DISADVANTAGES OF REDUCED SILVER IN DENTAL THERAPEUTICS *

BY U. GARFIELD RICKERT, M.A., D.D.S., ANN ARBOR, MICH.

This paper is offered at this time, because of the large number of inquiries made during the past year regarding the merits of the so-called silver reduction method as applied in dentistry. At the several district meetings at

* Read before the Michigan State Dental Society, April 7-11, 1919, and published in *Journal N. D. A.*

which we recently appeared, about the first question asked was for an expression regarding this treatment.

For a number of years we have been interested in reducing silver in different types of teeth, not then having in mind so much the possible therapeutic application as a study of the variation in physical constitution of the several tooth tissues. Naturally this information, with the general knowledge of the sterilizing properties of silver, and with the extraordinary tolerance of the tissues toward silver, added especial interest to the sweeping claims recently made by Dr. Percy Howe of Boston.

Many of you have tried this modern panaca and found it extremely irritating. We too made up the solutions as recommended and admit that nothing we ever blundered in gave us so much embarrassment as those days of suffering on the part of patient victims. To this date we hate the ring of a telephone because it recalls that voice, ''What shall I do, for I just can't stand this thing any longer?'' The fact of the matter was that there was nothing one could do but resort to general sedatives, for these irritations usually lasted from thirty-six to seventy-two hours, during which time the suffering of the patient was very severe.

Now we are in no way trying to minimize the importance of the conscientious work of Dr. Howe, for we recognize that he has pointed out the possibilities of silver in dental therapeutics and certainly few subjects have recently aroused the interest of the profession to a greater degree than the one of silver reduction. If we were to criticize the above efforts, which is not the purpose of this paper, our criticism would be simply this, that judging from our experience with it, it is too sweeping in its claim, somewhat misleading, and too much like magic to be true.

The recruits on the side of promiscuous extractions have been, during the past year, increased at an alarming rate; those of us who have been trying to keep in the middle of the road are grasping at every straw for more positive methods; many operators are willing to try anything—for this reason no method should be given unqualified approval until such method has been thoroughly tried. My own experience with Dr. Howe's method as suggested in his original paper is evidence of the harm that may come by giving out a misleading technic. Radiograms of the cases of extreme irritation of over a year's standing are not at all encouraging. Dr. Howe tells us that it is unnecessary to put any of these irritating solutions through the teeth. He infers that by placing a small amount into the pulp chamber, it will be carried just to the end of the canal and not through. My experience has been somewhat different, possibly due to less operative ability on my part.

We have not always been able in the mouth or in most extracted teeth where the apical foramina were sealed, to have absolute control of the end point. In situ, it will either not go quite to the end, or if carried into the canals with broaches, it will go through. Evidently then it was necessary to modify the treatment so that if a small amount did pass to the periapical tissue, it would not be so irritating. Unless we are able to completely fill canals to the end we are defeating the very purpose we desire. We might as well use any other filling material if filling as far as possible were sufficient. One need only examine the illustrations accompanying Dr. Howe's paper to observe that Dr. Howe filled to the end in extracted teeth, but some of those treated in the mouth show little or no silver at the apices.

For the benefit of those who are interested in knowing to what extent we have been able to verify Dr. Howe's seven applications, a brief discussion of the several claims will be given at this time. Quoting from the original paper —application number one: "It is effective in the sterilization of disintegrated dentin overlying pulps as in large cavities of carious first molars."

The treatment has been modified so that cases of a year's standing give promise of developing into a sound technic. After careful bacteriologic tests we know, at this time, of no surer or better way for the sterilizations of infected dentin. The technic used will be given later in this paper.

Application number two: "By this method it is possible to completely sterilize not only a putrescent pulp without removing it, but also the dentinal structure as well."

We have proven that it is possible to sterilize such pulps but for reasons to be given later we question its application in practice.

Application number three: "In acute pericementitis following the death of the pulp, it is only necessary to pump this material into the pulp chamber and into the canals as well as possible and close the tooth. The pericementitis in such cases as we have treated, has been quickly allayed and a subsequent and more thorough treatment has apparently ended the trouble."

We have been unable to substantiate this statement. Judging from the reports of an enormously large number of operators, it is easier to believe that it has caused more cases of pericementitis than it has allayed.

Application number four: "We have found it effective in the treatment of chronic abscesses."

We have learned from bacteriologic test and clinical observation, in both temporary and permanent teeth that silver is not well adapted to sterilization of periapical tissue, other treatment should be used and silver depended upon to fill the apical foramina and sterilize the dentin.

Application number five: "We believe this to be an admirable means of taking care of apical foramina in all cases if properly used."

Our findings have been in accord with the first part of this statement, but we are not ready to justify its use in all cases as suggested in the second part of claim five.

Application number six: "It is an excellent means for almost painlessly removing the remaining part of a pulp, after the death or removal of a portion of it."

As to this statement we have little to say for we have no occasion indicating its use. It will probably accomplish what is claimed for it, especially if the reducing agent is formalin. Formalin and certain of its compounds will do the same thing. We question, however, the promiscuous use of such drastic treatment in uninfected teeth.

Dr. Howe's seventh claim: "Applied to the root ends after apioectomy it lessens the probability of subsequent trouble."

A number of cases of a year's standing under our observation are evidence that if properly applied there are several good reasons for this procedure. A discussion of the technic of the same will be taken up later.

Our conclusions regarding the several silver treatments are based, first, on some well-established therapeutic and bacteriologic principles. Second, on clinical observation. Third and last, laboratory experiment. It will be evident that the time for clinical observation has been limited. Our cases of longest standing are only of fifteen months' duration. Years only will verify whether or not our deductions are absolutely sound.

This is a day of preventive dentistry; for this reason the preventive applications of reduced silver will be considered first. The simplest conditions indicating silver for prophylactic reasons are areas susceptible to caries in temporary teeth. We next have the small cavities where it is impossible to do ideal work. In such cases one may, by a little care, inhibit further development of caries. You have all seen small cavities into which silver has been reduced years previous, in which there has been no further decay. In tomorrow's clinic sections of teeth will be exhibited, showing the resistance toward weak acids where dental structures have been impregnated with reduced silver, as compared with untreated sections. Now, in the treatment of these conditions, the ammoniacal solutions offer no special advantage over an ordinary silver nitrate solution.

Likewise sections of teeth will be exhibited intending to demonstrate that the latter is even to be preferred because of its greater penetration in conditions where the protoplasmic processes of the dentinal tubules have been destroyed; where this is not true the amoniacal solutions penetrate farther. Silver nitrate is less easily reduced from its cold solutions than the ammoniacal solutions and consequently penetrates further in dentin before being reduced by the action of light and organic matter, providing the protoplasmic processes have been destroyed. We have repeatedly demonstrated in our studies of permeable membranes, the impermeability of dental tissues in which there has been a reduction of silver. Dentin and enamel thus treated become clogged with silver, consequently are not so easily affected by chemical action.

Before giving details of technic of the several applications where reduced silver has in our hands given a measure of success, several terms to be used later will be defined.

### SLOW REDUCTION

By this term you are to understand that a number of days are required before reduction to the metallic state is complete. By rapid reduction we imply that the reduction is more or less complete in a few minutes. In our experience there are indications for the use of one or the other of these distinctive types of reduction.

A few years ago it was our practice either to devitalize or to extract teeth from which we were unable to remove all decay because of proximity to the pulp. For reasons which need no discussion here, we are making more strenuous efforts for the conservation of healthy pulps. The treatment we are about to describe for the above condition has been applied in a large number of cases. Pulps of this kind treated fifteen months ago that were recently tested were found to be in excellent condition. Results of a large number of more recent treatment are also very encouraging. The treatment used is as follows: First apply the rubber dam, then prepare the cavity so that there is a sound enamel wall. Remove soft and infected dentin, avoiding too near exposure of the pulp. The dentin must be cut so that there will be some firm dentin in the line of force of mastication for the cement to rest upon. The cavity is dried avoiding excessive heat. The cavity is then ready for the rapid reducing silver, the preparation of which will be described later. By a little care the silver may be applied without discoloring the margins. Eugenol, or even dentalone, may be used as the reducing agent which should be applied several minutes after the silver solution. The excess should be removed and the treatment repeated several times. The excess is again removed and the wall in close proximity to the pulp is slightly moistened with eugenol and this area is covered with a thin layer of dry zinc oxide which is easily held in position by the moist surface. The reason for the latter is twofold: It insures complete reduction of the silver near the pulp. This shortens the time, which is very essential in serious irritation. The zinc oxide admits of a thinner mix of cement, consequently close adaptation is made possible without pressure to the frail pulpal wall. There is no irritation because of excess phosphoric acid, for such excess is taken care of by the layer of zinc oxide. After capping with the cement, the cavity is ready for whatever restoration is desired.

While we are not giving this treatment as a finished method, it is safe to say that results of a large number of cases for the limited time we have had in which to observe such results have been very satisfactory. Careful tests should be made to make certain that there has been no serious degeneration of pulp preceding this treatment. If formalin is used for the reducing agent, it must be very dilute and enough silver solution must be used to insure complete decomposition of the formalin. The cavity should be treated with sodium bicarbonate to neutralize the acidity following the reduction with formalin. This, however, is unnecessary where essential oils are used as the reducing agent.

The silver solution used for rapid reduction is made up as follows: Silver oxide is precipitated from a silver nitrate solution with KOH or NaoH. This is carefully washed to remove all impurities and kept moist in a small amber-colored bottle. In this condition reduction is so slight that we have kept it for a long time without much damage. If a small amount is insoluable in excess of ammonia, there has been too much reduction and the silver oxide should be freshly prepared. This is our stock solution and it will be seen that it is more desirable than the ammoniacal solution made from silver nitrate, because it is free from nitric acid and other impurities.

Now when we desire rapid reduction as in a pulp capping or root resection, the silver oxide is added to a drop or two of ammonium hydroxide to the point of saturation. In this state the ammoniacal solution is easily reduced. The ease with which reduction takes place with this solution is so marked that even burnishing with a warm glass rod is sufficient to reduce to the lustrous metallic silver. There is one precaution that must be mentioned here. That is, that after a few minutes fulminates of silver may be formed from the ammoniacal solutions, which are very explosive. I have taken old solutions of both Dr. Howe's and my formulas and incompletely reduced the same in teeth or on blotting paper, both producing explosions on being touched with a hot platinum wire or by use of the electric current. Serious accidents are possible from even wet solutions. Only very small amounts of ammoniacal solutions should be made and the unused portion discarded immediately after treatment. After complete reduction there is no danger and only ammoniacal solutions, especially after drying, are to be avoided.

The filling of apical foramina deserves especial attention. The possibility of filling difficult canals with reduced silver, providing the irritating properties were eliminated, prompted investigation as to the cause of irritation. The following experiment is an explanation of the probable cause. An ammoniacal solution of silver was reduced with formalin, another with eugenol. The freshly reduced silver is filtered and reaction of the filtrate tested. The filtrate from the formalin reduction is at first strongly acid, but on standing a few hours or on heating the acidity is diminished.

Now if by the aid of a needle, a little of this filtrate is passed beneath the skin, it will produce a smarting sensation not unlike the bite or sting of certain insects. Since formic acid has been demonstrated in the secretions attending such stings, one would be inclined to suspect formic acid in the above mentioned filtrate. The filtrate where eugenol is the reducing agent does not produce the smarting sensation. Time did not admit of more elaborate tests and the specific acid is by no means proven by this simple test. The experiment was evident that the amount of formalin should be diminished far below the amount suggested in Dr. Howe's paper. In a large number of cases in which the formalin was diminished and eugenol or dentalone and sodium carbonate were substituted, there was no irritation. That there are objections to this technic must be evident because we are introducing too many elements not silver. We are aware of the fact that elimination of the formalin probably diminishes the antiseptic properties. A large number of cases do not require so powerful an antiseptic. The dentin may be filled equally well with the rapid reducing method suggested for nearly exposed pulps without the serious irritation which we have learned to fear more than a few undestroyed bacteria in the remote recesses of the dentin. One advantage of reduced silver for canal fillings over other methods is that it does away with the danger of over-instrumentation to accomplish complete filling. There is a reason to believe that over-instrumentation has been the cause of a great deal more trouble than most operators are aware of. It is unnecessary, if reducing silver is used, to work as close to the apex as required by other methods.

Then ammonia has also been the cause of some of the irritation. There is no doubt that careful preparation of the solutions will prevent this. Infected periapical areas should be treated by methods that have given seemingly good results in the hands of the individual operator. The dentin and apical foramina may be filled with silver after careful cleaning of the canals. Short-softened pieces of gutta-percha points are then packed into the enlarged portion of the canal. Where more drastic treatment is required than is possible by the above method, surgical aid, either extraction or root-resection, seems to be the only avenue of escape at the present time. Numerous tests have demonstrated that silver and formalin will, if properly used, sterilize the canals and dentin of infected roots; but that this sterilization is limited to the tooth and not to tissues far beyond the root has also been repeatedly demonstrated. We have on exhibition a tooth recently extracted in which the canals were filled fifteen years ago with copper. There was no discomfort or evidence of infection. The crown had broken down so that only the copper impregnated roots remained. This is a fine illustration of the tolerance of tissues to copper. The roots were jet black, resisting for fifteen years the action of body fluids. What copper will do we believe silver will do better.

The possibilities of reduced silver in the preparation of roots for resections seem to give most encouraging results. The following technic is one one used preparatory to resection: The canals are opened, using the rubber dam where this is possible, and the silver solution is introduced to the very apex. Five or ten minutes are then allowed for penetration before the reducing agent is applied. This treatment is applied several times. There is no objection to the use of Dr. Howe's solutions for this operation, because the endangered apical tissue is to be excised immediately following the filling of the canals. The canals are very carefully packed with small pieces of gutta-percha points softened by gently heating. The root is resected and the resected end dried as well as possible and silver, by rapid reduction, is reduced and burnished over the resected end. The latter treatment is essential because dentin is poorly adapted to resist the action of body fluids, and experimentally at least, this procedure prevents resorption. Then too the silver prevents the exposed softened dentin from becoming infected in the healing process and to later renew the trouble. It also forms another barrier to subsequent infection from the root-canal. It will be evident that after healing of such resections it becomes difficult to have patients submit to the necessary means for obtaining material for bacteriologic tests. We have made but one such test and that was found to be sterile. While this proves nothing, it is at least favorable evidence.

The necessary length of this paper prevented the addition of the chemical and bacteriologic tests made in investigating the possible uses of silver in dental therapeutics. These, with other methods that are under observation, will be presented in another paper to appear later.

### A SUMMARY

The possible advantages of reduced silver would suggest its use for prevention of caries, the protection of nearly-exposed pulps, the possibility of filling certain tortuous canals, and preparation and treatment in root-resection.

As to the disadvantages, the instability of the solutions, the discolorations attending its use, and possible irritations are the most important. While these disadvantages are subject to control, they offer no encouragement in root-canal work to operators desiring a method infallible in careless operations. The fact that silver solutions properly reduced will sterilize should not be depended upon to admit of less care than that required by other methods. We caution operators not to be over-confident in the sterilizing properties of silver and take too long chances. There is already evidence that this has too often been the case and serious results are certain to follow.

It is to be regretted that the substance of this paper could not be more constructive and less destructive, but unfortunately, this could not be avoided for the proposition as to the disposition of pulpless teeth is not an easy one. It is and has been seriously studied by a large number of men of genius and ability, and as yet no one has been able to offer us anything that is safer than extraction. We recently made a study of a large number of treated pulpless teeth which was convincing in two respects: first, that the evidence is overwhelmingly against poor root-canal surgery; and the second, that which at this time concerns us most is the fact that the evidence is not overwhelmingly against good root-canal surgery. In fact, it is evident that it is not an impossibility to safely treat devitalized teeth, and that we have failed only because we have not as yet a method of treatment that is easy enough of operation to be universally adopted in practice. Then there is but one course for the operator to pursue. That is to play safe and study each case carefully. If conditions are favorable and the skill of the operator is such that a perfect root filling can be inserted, this is certainly the better course. But if conditions are unfavorable and the operator realizes that he can not do a safe operation, then there is only one thing to do. That is to remove the tooth rather than jeopardize the general health of the patient. For not only the reputation of the individual operator, but that of dentistry, is at stake.

